# Molecular Insights of Copper Sulfate Exposure-Induced Nephrotoxicity: Involvement of Oxidative and Endoplasmic Reticulum Stress Pathways

**DOI:** 10.3390/biom10071010

**Published:** 2020-07-08

**Authors:** Chongshan Dai, Qiangqiang Liu, Daowen Li, Gaurav Sharma, Jianli Xiong, Xilong Xiao

**Affiliations:** 1 College of Veterinary Medicine, China Agricultural University, No. 2 Yuanmingyuan West Road, Beijing 100193, China; daichongshan@cau.edu.cn (C.D.); lidaowen@tjau.edu.cn (D.L.); 2 College of Animal Science and Technology, Henan University of Science and Technology, Luoyang 471023, China; liuqiang_1017@sina.com; 3 Advanced Imaging Research Center, University of Texas Southwestern Medical Center, Dallas, TX 75390, USA; Gaurav.Sharma@UTsouthwestern.edu

**Keywords:** copper, oxidative stress, apoptosis, endoplasmic reticulum stress, kidney, cytotoxicity

## Abstract

The precise pathogenic mechanism in Cu exposure-cause nephrotoxicity remains unclear. This study investigated the underlying molecular mechanism of copper sulfate (CuSO_4_)-induced nephrotoxicity. Mice were treated with CuSO_4_ at 50, 100, 200 mg/kg/day or co-treated with CuSO_4_ (200 mg/kg/day) and 4-phenylbutyric acid (4-PBA, 100 mg/kg/day) for 28 consecutive days. HEK293 cells were treated with CuSO_4_ (400 μM) with or without superoxide dismutase, catalase or 4-PBA for 24 h. Results showed that CuSO_4_ exposure can cause renal dysfunction and tubular necrosis in the kidney tissues of mice. CuSO_4_ exposure up-regulated the activities and mRNA expression of caspases-9 and -3 as well as the expression of glucose-regulated protein 78 (GRP78), GRP94, DNA damage-inducible gene 153 (GADD153/CHOP), caspase-12 mRNAs in the kidney tissues. Furthermore, superoxide dismutase and catalase pre-treatments partly inhibited CuSO_4_-induced cytotoxicity by decreasing reactive oxygen species (ROS) production, activities of caspases-9 and -3 and DNA fragmentations in HEK293 cells. 4-PBA co-treatment significantly improved CuSO_4_-induced cytotoxicity in HEK293 cells and inhibited CuSO_4_ exposure-induced renal dysfunction and pathology damage in the kidney tissues. In conclusion, our results reveal that oxidative stress and endoplasmic reticulum stress contribute to CuSO_4_-induced nephrotoxicity. Our study highlights that targeting endoplasmic reticulum and oxidative stress may offer an approach for Cu overload-caused nephrotoxicity.

## 1. Introduction

Copper (Cu) is an essential trace element required for the formation of different metalloenzymes (e.g., Cu, Zn-superoxide dismutase, cytochrome-c oxidase, ceruloplasmin and dopamine beta-mono-oxygenase) that play critical roles in a variety of biological processes including metabolism, nutrition, and development in human and animals [1]. The malfunctioned Cu homeostasis is intricately linked with human diseases and pathophysiologies [1]. Copper is also a common pollutant of the metals used in industrial and agricultural processes [2]. In China, copper sulfate (CuSO_4_) as a veterinary food additive has been widely used in animal production, this may lead to potential soil pollution and ecotoxicity [3]. During recent decades, Cu exposure or pollution-caused toxic effects and Cu overload-caused diseases (e.g., Wilson’s disease and neurodegenerative diseases) have posed substantial public health issues [4,5].

Copper can be transported into the cell by its special transporter (e.g., human copper transporter 1 in human cells) [6] and accumulated in various tissues or organs, including kidney, liver, heart, brain, and reproductive organs [1]. Copper ions can either exist as Cu1^+^ (reduced) or Cu^2+^ (oxidized) in biological redox reactions [7]. The excess free Cu is redox-active and catalyzes the production of hydroxyl radicals in a Cu-dependent ‘Fenton-like’ reaction, then triggering oxidative stress and damaging cells or tissues [1]. It has been reported that the high dose of Cu exposure can damage various tissues and organs, including the kidney, brain, liver, intestines, and testis [5,8,9,10,11,12,13,14]. In particular, the kidney is vulnerable to toxic effects from Cu due to its filtration functions. The pathogenic mechanisms of Cu-induced nephrotoxicity are not fully understood.

Copper-induced renal glomerular and tubular dysfunction is exacerbated by proteinuria, diminished glomerular filtration, amino aciduria, and renal phosphaturia [15]. Oxidative damage is one of the hallmarks in Cu exposure-induced toxicity [16]. Chronic CuSO_4_ exposure can induce mitochondrial dysfunction and cell apoptosis in chicken kidney tissues [17]. In addition, pathways for rapamycin (mTOR) mammalian target, autophagy activation and nuclear factor kappa B (NF-κB) may also be associated with CuSO_4_-induced nephrotoxicity [17,18]. Endoplasmic reticulum (ER) is an important organelle that plays a vital role in storing calcium, synthesizing proteins and detoxifying harmful substances [19]. ER stress-mediated apoptosis was detected in mouse liver tissue exposure to CuSO_4_ [8]. The previous studies have demonstrated that ER stress is affected by some drugs (e.g., colistin and cisplatin), toxins (e.g., zearalenone) and heavy metals (e.g., lead and cadmium)-induced renal damage in vitro and animal models [20,21,22,23,24,25]. ER stress-mediated apoptosis was observed in mouse liver tissues and involved the activation of growth arrest- and DNA damage-inducible gene 153 (GADD153/CHOP), c-Jun N-terminal kinase (JNK) and caspase-12 signaling pathways [8]. Up until now, the ER stress contribution in CuSO_4_ exposure-induced nephrotoxicity remains poorly understood. The present study therefore investigated the role of oxidative stress and ER stress in CuSO_4_-induced renal damage in a mouse model and also attempted to explore the underlying molecular mechanisms of toxicity in HEK293 cells.

## 2. Materials and Methods

### 2.1. Chemicals and Reagents

CuSO_4_·5H2O was purchased from Sinopharm Chemical Reagent Co., Ltd. (Shanghai, China). Sodium dodecyl sulfonate (SDS), aprotinin, leupeptin, pepstatin A, and phenylmethylsulfonyl fluoride (PMSF) were obtained from AMRESCO Inc. (Solon, OH, USA). Cell Counting Kit-8 (CCK-8) was purchased from Med. Chem. Express (Shanghai, China). Superoxide dismutase (SOD), catalase (CAT), and 4-phenylbutyric acid (4-PBA) (purity ≥ 98%) were purchased from Sigma-Aldrich (St. Louis, MO, USA). Dulbecco’s modified Eagle’s medium (DMEM) and fetal bovine serum (FBS) were purchased from Life Technologies Corporation (Grand Island, NY, USA). 2′,7′-dichlorofluorescein-diacetate (DCFH-DA) and 0.25% Trypsin-EDTA were purchased from Beyotime Biotechnology (Haimen, China). All other chemicals were of analytical grade.

### 2.2. Animals and Treatments

All animal experiments were approved (No.HNUST-2019-0401-01) by the Institutional Animal Care and Use Committee at the Henan University of Science and Technology (Luoyang, China). C57BL/6 mice (male, eight weeks old, 20–22 g) were purchased from Vital River Animal Technology Co., Ltd. (Beijing, China). An acclimation period of 1 week was employed before the experiments. Mice were given ad libitum access to food and water, and held in a room maintained at a temperature of 23 ± 2 °C and relative humidity of 50 ± 10% with a light–dark period of 12 h.

Forty mice were divided into the following 4 groups (*n* = 10 in each group): (1) control, (2) CuSO4 50 mg/kg/day, (3) CuSO_4_ 100 mg/kg/day and (4) CuSO_4_ 200 mg/kg/day, respectively. In CuSO_4_ groups, mice were orally administrated with CuSO_4_ at the dose of 50, 100 and 200 mg/kg/day for the following 28 days. Mice in the control group received an equal volume of vehicle.

To further investigate the role of ER stress on CuSO_4_-induced nephrotoxicity, thirty-two mice were divided into 4 groups (*n* = 8 in each group): control group, 4-PBA group, CuSO_4_ model group and 4-PBA + CuSO_4_ group. The mice were orally administrated 4-PBA at 100 mg/kg/day or CuSO_4_ 200 mg/kg/day, or 4-PBA at 200 mg/kg/day 2 h prior to CuSO_4_ at 200 mg/kg/day. The mice in the control group were administrated an equal volume of vehicle. All mice were consecutively treated for 28 days.

One day after the last dose, all mice were euthanized using an intraperitoneally administered overdose of sodium pentobarbital (80 mg/kg). Blood samples were centrifuged at 3000× *g* for 10 min (Sigma, Goettingen, Germany) for measurements of blood urea nitrogen (BUN) and serum creatinine (CRE). The kidney tissues were isolated and weighed. Then, one part of the kidney was immediately fixed in 10% formaldehyde for histopathology observation, and the rest was frozen with liquid nitrogen, and stored at −80 °C until it was used for biochemical, histopathological and gene expression examination, respectively.

### 2.3. Cell Culture

The human HEK293 cell line was purchased from Cell Bank of Type Culture Collection of Chinese Academy of Sciences (Shanghai, China) and cultured in DMEM medium with 10% (*v*/*v*) heat-inactivated fetal calf serum, 110 mg/L sodium pyruvate, 100 units/mL penicillin, and 100 μg/mL streptomycin. Cells were maintained in a humidified atmosphere of 95% air and 5% CO_2_ at 37 °C.

### 2.4. Measurement of BUN and Creatinine CRE

The levels of serum BUN and CRE were determined by using an automated chemical analyzer (Hitachi 7080, Hitachi High-Technologies Corporation, Tokyo, Japan) with the standard diagnostic kits (Shanghai Kehua Bio-engineering Co., Ltd., Shanghai, China).

### 2.5. Histopathology Examination

Three mice are randomly selected and the parts of a kidney from each mouse were fixed in 10% neutral buffered formalin for 48 h. The samples were de-waxed in xylene and rehydrated in a series of graded alcohols and then embedded in paraffin. The samples were sectioned at 4 μm and stained with hematoxylin–eosin (H&E) for light microscopic examination. The histopathological scoring was conducted, and renal tubular damage was evaluated using a semi-quantitative score (SQS) according to the previous study [20]. The final results were expressed as the SQS (mean ± standard deviation (SD)).

### 2.6. Measurement the Levels of Malondialdehyde (MDA) and Activities of Superoxide Dismutase (SOD), and Catalase (CAT)

Kidney tissues were homogenized at 4 °C in 9 volumes (approximately 1 mL per 0.1 g of tissue) of cold Tris buffer (0.01 M Tris-HCl, 0.1 mM EDTA-Na_2_, 0.01 M sucrose, 0.9% saline; pH 7.4). The resultant homogenates were centrifuged (14,000× *g*) at 4 °C for 15 min. The levels of MDA and the activities of SOD and CAT in the kidney homogenates by using commercial assay kits (Nanjing Jiancheng Bio-Corporation, Nanjing, China). The protein content was measured by using a BCA protein assay kit (Nanjing Jiancheng Bio-Corporation, Nanjing, China).

### 2.7. qRT-PCR

The expressions of glucose-regulated protein 78 (GRP78), GRP94, CHOP, caspase-12, caspase-3, caspase-9, and Bax mRNAs were measure using an AB7500 real-time PCR instrument (Applied Biosystems, Foster City, CA, USA). Briefly, the total RNA was isolated from kidney tissue using TRIzol^®^ reagent (Life Technologies, Grand Island, NY, USA). The cDNA was synthesized from 1 μg RNA using the Prime Script RT-PCR kit (Takara, Dalian, China). The quality of RNA was ascertained by measuring the OD at 260/280 nm. The primer sequences (5′-3′) used are documented in Table 1. Target gene expression levels were normalized to the housekeeping gene GAPDH.

### 2.8. Measurement of Caspase-3, -9 Activities and DNA Fragmentation

The activities of caspase-3 and -9 in cells or kidney tissues were measured by using the Assay Kit according to the manufacturer’s instructions (Beyotime, Beijing, China). Protein concentrations were measured using a BCA™ protein assay kit (Beyotime, Beijing, China). The values of the activities of caspases-9 and -3 were normalized based on the protein content.

The DNA fragmentation examination is based on measuring the amount of mono- and oligonucleosomes in the cytoplasmic fraction of tissue extracts using a commercially available kit (Roche Diagnostics, Indianapolis, IN, USA) according to manufacturer’s instructions.

### 2.9. Measurement of Cell Viability

HEK 293 cells were seeded in a 96-well plate at a density of 2 × 10^4^ cells/well in 100 μL of culture medium for 16 h. Cells were incubated with the different concentrations of CuSO_4_ (25, 50, 100, 200, and 400 μM) for 24 h. After treatment, a fresh medium containing 10 μL CCK-8 solution was added into each well of the plate. Incubate the plate for 1 h in the incubator. The absorbance was read using a microplate reader at 450 nm (Tecan Trading AG, Männedorf, Switzerland).

To investigate the role of oxidative stress or ER stress on CuSO_4_-induced cytotoxicity, cells were pre-treated with SOD at 2 μg/mL or CAT at 10 μg/mL for 1 h or 4-PBA at 0.5, 1 and 2 mM for 2 h before CuSO_4_ treatment (400 μM). As a positive control, HEK 293 cells were pre-treated with 4-PBA at the concentration of 2 mM for 2 h, followed to treat with tunicamycin at 4 μg/mL according to the previous study [26]. After an additional 24 h, the cell viability was measured.

### 2.10. Measurement of Reactive Oxygen Species (ROS)

The peroxide production was measured using the fluorescent probe 2,7-dichlorofluorescein diacetate (DCFH-DA) staining cells as previously stated [27]. DCFH-DA was diluted with a serum-free medium at 1:1000, and the final concentration was 10 μmol/L. After treatment, the cell culture medium was removed and replaced with 1 mL DMEM containing DCFH-DA and incubated for 20 min at 37 °C in the dark. After three washes with a serum-free medium, the DCFH-DA fluorescence was observed by a fluorescence microscope.

### 2.11. Statistical Analysis

All data are presented as mean ± SD. All figures were drawn by using Graph Pad Prism 8.2 (Graph Pad Software, Inc., San Diego, CA, USA). A one-way analysis of variance, accompanied by a Tukey’s multiple comparisons test, was used to compare any two means when the variance was homogeneous, otherwise, Dunnett’s T3 test was used (Graph Pad Prism 8.2.). A *p*-value (<0.05) was considered statistically significant.

## 3. Results

### 3.1. CuSO_4_ Exposure Induces Renal Dysfunction and Tubular Necrosis in Mice

**Figure 1:**
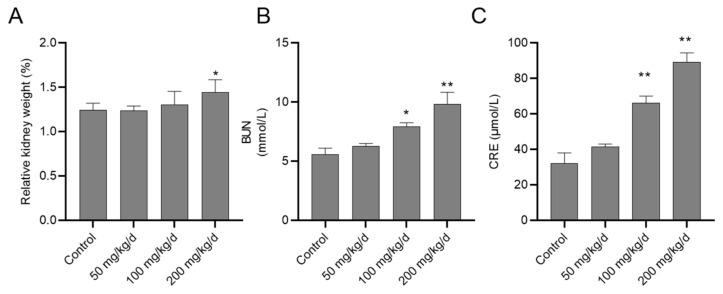
The changes of relative kidney weight (**A**) and the levels of serum blood urea nitrogen (BUN) (**B**) and serum creatinine (CRE) (**C**) in mice exposed to copper sulfate (CuSO_4_). The mice were orally administrated with copper sulfate (CuSO_4_) at the dose of 50, 100 and 200 mg/kg/day for 28 days, the relative kidney weight, BUN and CRE were examined. Data are presented as mean ± SD (*n* = 10). * *p* < 0.05, or ** *p* < 0.01, compared to the control group.

The body weights in mice were unchanged after CuSO_4_ exposure at 50, 100 and 200 mg/kg/day for 28 days, compared to the control group, however, the relative kidney weight significantly increased in the CuSO_4_ 200 mg/kg/day group (*p* < 0.05) (Figure 1A). In the CuSO_4_ 100 and 200 mg/kg/day groups, serum BUN significantly increased to 7.91 (*p* < 0.05) and 9.86 mmol/L (*p* < 0.01), respectively (Figure 1B); serum CRE significantly increased to 66.14 and 89.28 μmol/L (both *p* < 0.01), respectively (Figure 1C). In the CuSO_4_ 50 mg/kg/day group, the serum BUN and CRE slightly increased, but had no significant difference, compared to that in the untreated mice.

**Figure 2:**
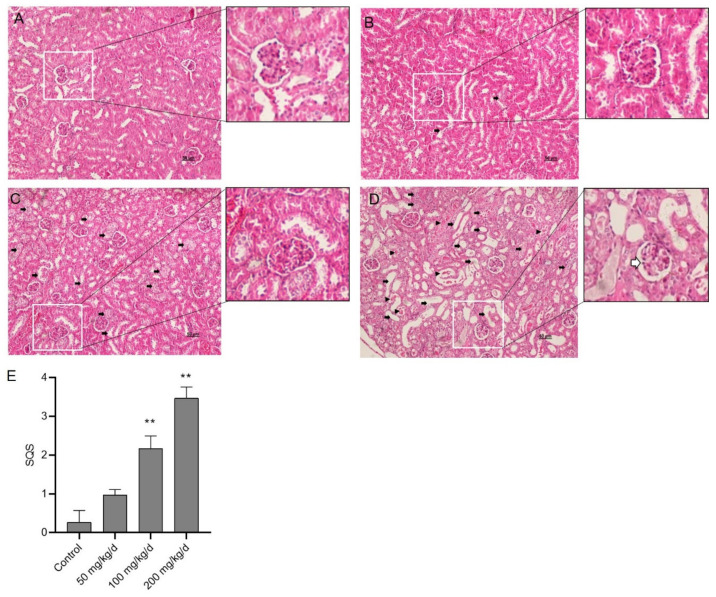
Representative histopathological changes in kidneys of mice and the semi-quantitative scores (SQSs). (**A**) Control group: no significant damage. (**B**) CuSO_4_ 50 mg/kg/day group: mild damage. (**C**) CuSO_4_ 50 mg/kg/day group: intermediate damage. (**D**) CuSO_4_ 200 mg/kg/day group: extensive damage. (**E**) SQSs are presented as the mean ± SD (*n* = 3). ** *p* < 0.01, compared to the control group. Filled arrows indicate tubular degeneration, necrosis and tubular dilation, arrowheads indicate cast formation, and the open arrow indicates glomerular abnormalities. Hematoxylin–eosin staining. Bar = 50 μm.

The histopathological examination of kidney tissues showed that CuSO_4_ exposure caused tissue damage in a dose-dependent manner (Figure 2). Compared to the control group, the kidneys of mice in the CuSO_4_ 50 mg/kg/day group showed the mild tubular damage (Figure 2B), the SQS value is 0.96 ± 0.25 (*p* > 0.05). Severe histological features of renal damage (e.g., tubular degeneration, necrosis, tubular dilation, cast formation, and glomerular degeneration) were detected in the CuSO_4_ 100 and 200 mg/kg/day groups, the corresponding SQS values are 2.17 ± 0.57 and 3.45 ± 0.29 (both *p* < 0.01), respectively (Figure 2C–E).

### 3.2. CuSO_4_ Exposure Causes Oxidative Stress Damage in the Kidney Tissues

**Figure 3:**
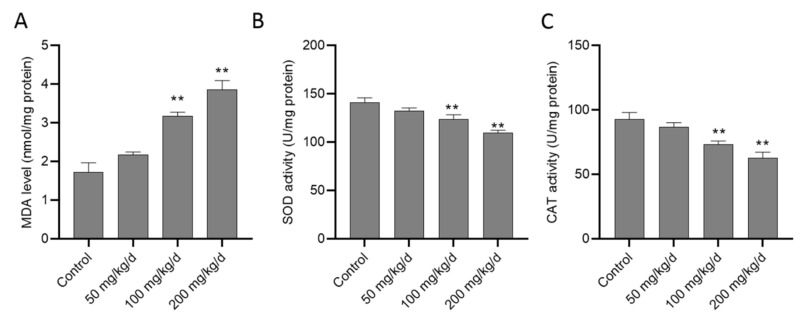
CuSO_4_ exposure induces oxidative damage in the kidney tissues of mice. The mice were orally administrated with copper sulfate (CuSO_4_) at the dose of 50, 100 and 200 mg/kg/day for 28 days, the level of malondialdehyde (MDA) (**A**) and the activities of superoxide dismutase (SOD) (**B**) and catalase (CAT) (**C**) in the kidney tissues were analyzed. Data are presented as mean ± SD (*n* = 7). ** *p* < 0.01, compared to the control group.

The biomarkers of oxidative stress in the kidney tissue were examined. As shown in Figure 3, the levels of MDA increased to 2.17 ± 0.20, 3.19 ± 0.26 (*p* < 0.01), and 3.87 ± 0.60 nmol/mg protein (*p* < 0.01) in groups of CuSO_4_ 50, 100 and 200 mg/kg/day (Figure 3A), respectively, compared to the control group. On the contrary, the activities of antioxidant enzymes SOD and CAT significantly decreased in the CuSO_4_ 100 and 200 mg/kg/day groups (all *p* < 0.01), respectively, compared to that in the control group. There was a slight decrease in SOD and CAT activities in the CuSO_4_ 50 mg/kg/day group compared to the control group (Figure 3B,C).

### 3.3. CuSO_4_ Exposure Up-Regulates the mRNA Expression of Bax, Caspases-3 and -9 as Well as the Activities of Caspase-9 and -3 and DNA Fragmentation in the Kidney Tissues

**Figure 4:**
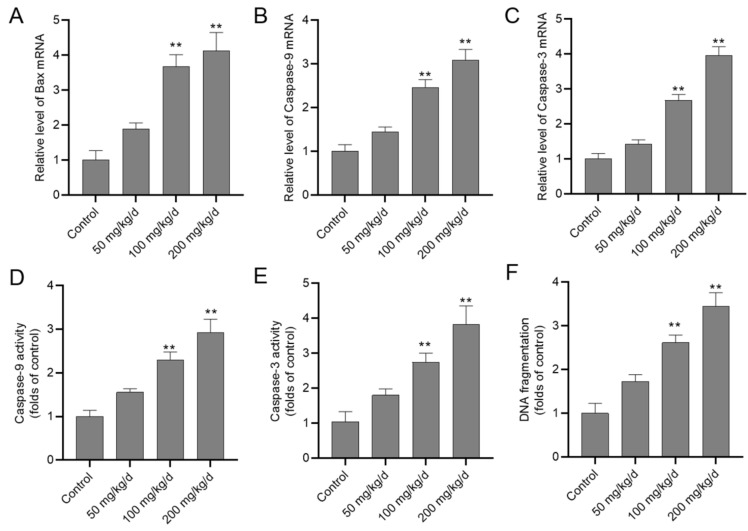
CuSO_4_ exposure activates the mitochondrial apoptotic pathway in the kidney tissues of mice. The mice were orally administrated with copper sulfate (CuSO_4_) at the dose of 50, 100 and 200 mg/kg/day for 28 days, the mRNA expressions of Bax (**A**), caspase-9 (**B**), and -3 (**C**) and the activities of caspase-9 (**D**), and -3 (**E**) in the kidney tissues were determined. The DNA fragmentation in the kidney tissues (**F**) was also examined. Data are presented as mean ± SD (*n* = 7). ** *p* < 0.01, compared to the control group.

As shown in the Figure 4, the expressions of Bax, Caspase-9 and -3 mRNAs significantly increased in the CuSO_4_ 100 and 200 mg/kg/day groups (all *p <* 0.01); the Bax, Caspase-9 and -3 mRNAs increased to 4.1-, 3.1- and 3.9-fold in the CuSO_4_ 200 mg/kg/day group, respectively, compared to the control group. Consistently, the activities of caspase-9 and -3 in the kidney tissues after mice were exposed with CuSO_4_ in a dose-dependent manner (Figure 4D,E). Compared to the control group, the activities of caspase-3 and -9 increased to 2.9- and 3.8-fold (both *p* < 0.01) in the CuSO_4_ 200 mg/kg/day group, respectively. Significantly increased DNA fragmentation in the kidney tissues of mice who are in the CuSO_4_ 100 and 200 mg/kg/day group (increased to 2.6- and 3.5- fold, respectively; both *p* < 0.01) were also detected.

### 3.4. Inhibition of Oxidative Stress Attenuates CuSO_4_ Exposure-Induced Cell Death in HEK293 Cells

**Figure 5:**
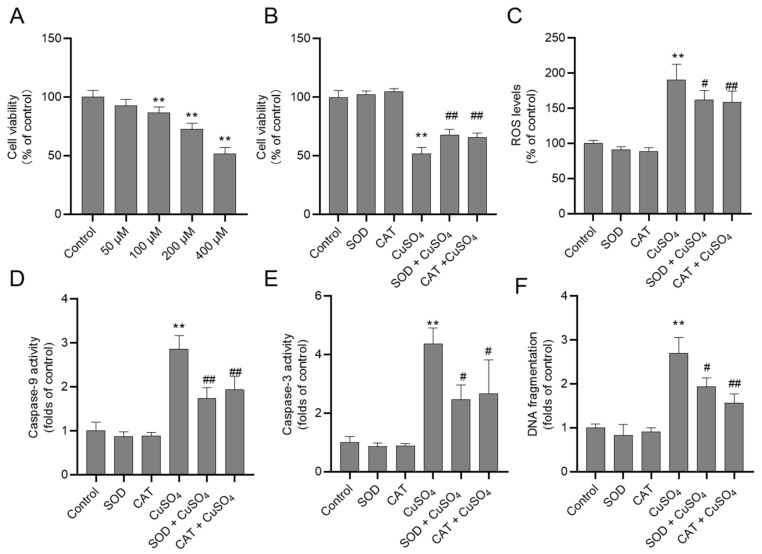
Changes of the cell viability, reactive oxygen species (ROS) production and the activities of caspase in HEK293 cells. (**A**) CuSO_4_ exposure at the dose of 50–400 μM induces the decreases of cell viability in a dose-dependent manner. (**B**–**E**), pre-treatment with superoxide dismutase (SOD), and catalase (CAT) inhibit CuSO_4_ exposure-induced decreases of cell viability (**B**), the production of ROS (**C**), the up-regulation of caspase-9 (**D**) and -3 (**E**) activities and increases of DNA fragmentation (**F**). Values were from three independent experiments and presented as mean ± SD (*n* = 3). ** *p* < 0.01, compared to the control group. ^#^ *p* < 0.05, or ^##^ *p* < 0.01, compared to the CuSO_4_ alone treatment group.

In HEK293 cells, CuSO_4_ exposure at the dose of 50–400 μM for 24 h induced the decrease of cell viability in a dose-dependent manner. As shown in Figure 5A, cell viability in HEK293 cells exposed to CuSO_4_ at the dose of 100, 200 and 400 μM (all *p* < 0.01) decreased to 86.7%, 72.9%, and 51.8%, respectively, relative to that in untreated cells. Pre-treatment of antioxidants SOD and CAT attenuated CuSO_4_ exposure (400 μM)-induced cytotoxicity; the cell viabilities increased to 67.7% and 65.8% (both *p* < 0.01), respectively, compared to CuSO_4_ alone treatment group (Figure 5B). Compared to the control group, CuSO_4_ exposure at 400 μM for 24 h, the level of ROS increased to 191.4% (all *p* < 0.01); the activities of caspases-9 and -3 increased to 2.9- and 4.4-fold (both *p* < 0.01) (Figure 5C–E), respectively. SOD and CAT pre-treatment partly decreased the production of ROS (decreased to 162.1% and 158.4%, respectively; *p* < 0.05 or 0.01), and the activities of caspase-9 (decreased to 1.7- and 1.9-fold, respectively; both *p* < 0.01) and caspase-3 (decreased to 2.5- and 2.7-fold, respectively; both *p* < 0.05), compared to CuSO_4_ alone treatment group. Similarly, SOD and CAT pre-treatment partially decreased the levels of DNA fragmentation (Figure 5F) compared to CuSO_4_ alone treatment group. There were no significant changes in the levels of cell viability, the production of ROS, caspase-9 and -3 activities, and DNA fragmentation in the SOD and CAT alone treatment groups, compared to the control group (Figure 5B–F).

### 3.5. CuSO_4_ Exposure Up-Regulates the Expression of GRP78, GRP94, CHOP and Caspase-12 mRNAs

**Figure 6:**
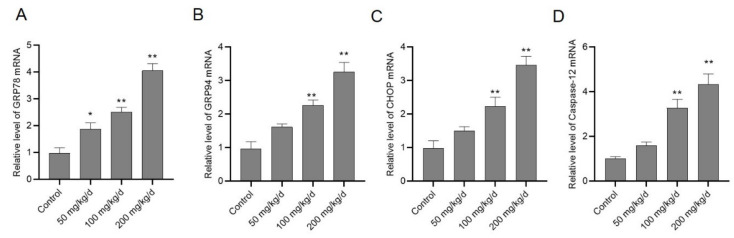
CuSO_4_ exposure activates endoplasmic reticulum (ER) stress in the kidney tissues of mice. The mice were orally administrated with copper sulfate (CuSO_4_) at the dose of 50, 100 and 200 mg/kg/day for 28 days, the mRNA expressions of GRP78 (**A**), GRP94 (**B**), CHOP (**C**) and caspase-12 (**D**) in the kidney tissues were determined. Data are presented as mean ± SD (*n* = 7). * *p* < 0.05, or ** *p* < 0.01, compared to the control group.

As shown in Figure 6, the expression of GRP78, GRP94, CHOP, and caspase-12 mRNAs were examined in the kidney tissues of mice after CuSO_4_ exposure. The increased mRNAs level of these genes after CuSO_4_ exposure presented in a dose-dependent manner (Figure 6). Correspondingly, compared to the control, the expression of GRP78, GRP94, CHOP, and caspase-12 mRNAs increased 4.1-, 3.3-, 3.5-, and 4.3-fold (all *p* < 0.01) in the CuSO_4_ 200 mg/kg/day group, respectively. These mRNA expressions had significant changes except for GRP78 (increased to 1.9-fold, *p* < 0.05) in the CuSO_4_ 50 mg/kg/day group, compared to those in the control group.

### 3.6. 4-PBA Pre-Treatment Attenuates CuSO_4_ Exposure-Induced Nephrotoxicity in Mice and Cytotoxicity in HEK293 Cells

**Figure 7:**
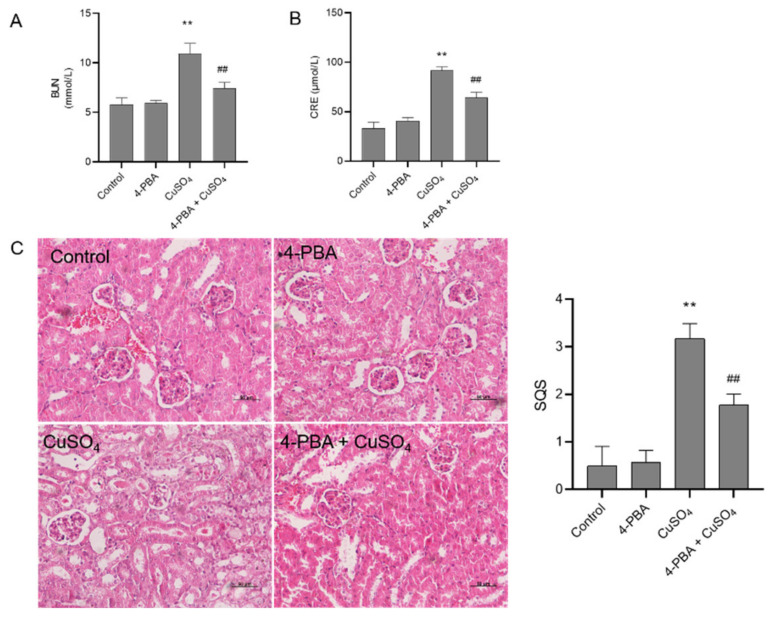
The protective effect of 4-phenylbutyric acid (4-PBA) administration on CuSO_4_ exposure-induced nephrotoxicity. (**A**,**B**) The levels of serum BUN and CRE, respectively. Data are presented as the mean ± SD (*n* = 8). (**C**) Representative histopathological changes (left) in kidneys of mice and the semi-quantitative scores (SQSs) (right). SQSs are presented as the mean ± SD (*n* = 4). ** *p* < 0.01, compared to the control group. ^##^ *p* < 0.01, compared to the CuSO_4_ group. Hematoxylin–eosin staining. Bar = 50 μm.

As shown in Figure 7, 4-PBA administration at 100 mg/kg/day markedly improved CuSO_4_ exposure-induced renal dysfunction. Compared to CuSO_4_ alone group, the levels of BUN and CRE in the CuSO_4_ + 4-PBA group significantly decreased from 10.9 mmol/L and 91.8 μmol/L to 7.4 mmol/L and 64.5 μmol/L (*p* < 0.01), respectively. Consistently, in the 4-PBA + CuSO_4_ group, the value of SQS decreased to 1.8 (*p* < 0.01), compared to the CuSO_4_ model group (the value of SQS is from 3.2). There were no marked changes in the serum BUN and CRE and the values of SQSs in the 4-PBA group, compared to untreated mice.

**Figure 8:**
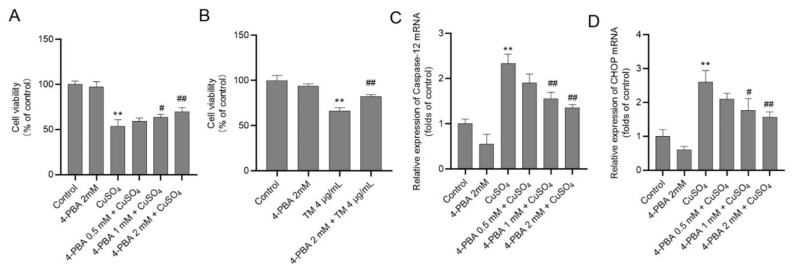
4-phenylbutyric acid (4-PBA) inhibits CuSO_4_ exposure-induced cytotoxicity and endoplasmic reticulum (ER) stress in HEK293 cells. (**A**) 4-PBA pre-treatment at the doses of 0.5, 1, and 2 mM inhibited CuSO_4_ exposure (at 400 μM)-induced cytotoxicity in a dose-dependent manner. (**B**) 4-PBA pre-treatment at the dose of 2 mM inhibits tunicamycin (TM, 4 μg/mL)-induced cytotoxicity. (**C**,**D**) The expression levels of caspase-12 and CHOP mRNAs, respectively. Values were from three independent experiments and presented as mean ± SD (*n* = 3). ** *p* < 0.01, compared to the control group. ^#^ *p* < 0.05, or ^##^ *p* < 0.01, compared to the CuSO_4_ alone or tunicamycin alone treatment groups.

Meanwhile, we examined the protective effect of 4-PBA on CuSO_4_ exposure-induced cytotoxicity. As shown in Figure 8, 4-PBA pre-treatment at 0.5, 1 and 2 mM for 2 h partly inhibited CuSO_4_ treatment-induced the decrease of cell viability (Figure 8A). Meanwhile, 4-PBA pre-treatment at 2 mM markedly inhibited ER stress inducer tunicamycin-induced cytotoxicity; the cell viability increased from 66.4% (i.e., tunicamycin alone group) to 82.2% (i.e., 4PBA + tunicamycin group) in HEK293 cells (Figure 8B). Furthermore, 4-PBA treatment at 1 and 2 mM significantly decreased the expression of caspase-12 and CHOP mRNAs (Figure 8C,D), compared to the CuSO_4_ alone treatment group.

## 4. Discussion

Copper pollution or poisoning can cause the damage of multiple organs (e.g., liver, kidney, and brain) of humans and animals [4,11,12,28,29,30]. Excessive intracellular levels of Cu can cause the imbalance of redox and result in a series of toxic events, such as cell apoptosis, necrosis and inflammation [31]. For example, in a rat model, oral administration of CuSO_4_ at the doses of 100 mg/kg/day and 200 mg/kg/day for 30 days could cause mild congestion in the glomeruli, focal congestion, and vacuolar degeneration of tubular cells in the kidneys of rats, and the renal damages are more serious with the extension of oral administration period of CuSO_4_ (e.g., 60 days or 90 days) [11]. In the present study, in a mouse model, oral administration of CuSO_4_ at 50, 100 and 200 mg/kg/day for 28 days, the relative kidney index (significant increases in the CuSO_4_ 200 mg/kg/day group; *p* < 0.05), serum BUN and CRE levels increased in a dose-dependent manner (Figure 1). Significant pathological damage was observed in kidneys from CuSO_4_ 100 and 200 mg/kg/day groups were observed, which showed tubular degeneration, necrosis, tubular dilation, cast formation and glomerular degeneration (Figure 2). These observations indicated that CuSO_4_ exposure can induce marked nephrotoxicity. Furthermore, the molecular mechanism was investigated in kidney tissues and HEK293 cells, and oxidative stress, mitochondrial dysfunction and ER stress (Figure 3, Figure 4, Figure 5, Figure 6, Figure 7 and Figure 8) were confirmed to partly contribute to CuSO_4_ exposure-induced nephrotoxicity.

The ability of copper to cycle in the body between the oxidized state of Cu (i.e., Cu^2+^) and reduced state of Cu (i.e., Cu^+^) is used by cuproenzymes that participate in redox reactions and many signaling pathways [7]. The transitions between Cu^2+^ and Cu^+^ can result in the generation of ROS, including superoxide anions and hydroxyl radicals, which could be removed by the intracellular antioxidants SOD), CAT and glutathione under physiological conditions [31,32]. An imbalance between the production of ROS and the antioxidant system defense will result in excessive ROS production, leading to damaging effects on lipids, proteins, and DNA, and ultimately cause cell death [33,34]. The previous study showed that after oral administration of CuSO_4_ 100 mg/kg/day and 200 mg/kg/day for 30 days, substantial increases in the level of MDA and decreases in total antioxidant potential in the liver, renal and brain tissues were observed in rats [12]. In another study, significant decreases in the SOD activities and levels of GSH were observed in the jejunum tissues of chickens fed with the basal diet plus 300 mg/kg CuSO_4_ for 30 days [35]. MDA is used as a biomarker of oxidative stress to evaluate the degree of the peroxidation of membrane lipids [36]. SOD can catalyze the dismutation of superoxide anion into oxygen and H_2_O_2_, which was further catalyzed into the water and oxygen by CAT [34]. In the present study, significant increases in MDA levels in the kidneys were detected in the CuSO_4_ 100 mg/kg/day and 200 mg/kg/day groups (Figure 3). Besides, we also observed a decrease in the activity of the antioxidant enzymes SOD and CAT in the kidneys of mice in the CuSO4 100 mg/kg/day and 200 mg/kg/day groups (Figure 3). Thus, these findings show that oxidative stress plays a significant role in CuSO_4_-induced nephrotoxicity in mice.

Mitochondria are the most vulnerable ROS targets and mitochondrial dysfunction can cause cell death by triggering apoptotic endogenous cascade reactions [37,38]. Caspase-3 is a key biomarker of apoptosis, which can be activated by both intrinsic and extrinsic apoptotic pathways and consequently lead to DNA breakdown [39]. Previous studies showed that mitochondrial dysfunction contributed to CuSO_4_-induced apoptosis in hepatocytes of chicken and rats and male germ cell line GC-1 cells [40,41,42]. The Bax up-regulation can promote the activation of mitochondrial permeability transition pores that mediated the release of cytochrome c (CytC) into the cytosol. CytC in the cytosol also leads to apoptosome formation, which triggers caspase-9 and caspase-3-mediated apoptosis [43]. It has been demonstrated that CuSO_4_ treatment significantly up-regulated the expression of Bax, CytC, caspase-9, and caspase-3 mRNAs and proteins followed to cause the cell apoptosis in chicken hepatocytes and male germ cells [44,45]. Rapamycin can partly inhibit mitochondria dysfunction (e.g., up-regulate the adenosine triphosphate (ATP) levels, mitochondrial mass, and mitochondria membrane potential) and attenuate CuSO4-induced cell apoptosis in chicken hepatocytes in vitro [41]. Consistent with previous studies, our current data showed that CuSO_4_ exposure significantly up-regulated the mRNA expression of Bax, caspase-9, and caspase-3 and the activities of caspase-9 and caspase-3 (Figure 4). Consistently, in the present study, CuSO_4_ exposure also significantly up-regulated the levels of DNA fragmentation in kidney tissues and HEK293 cells (Figure 4F and Figure 5F). Similar to caspase-3, DNA fragmentation is also a hallmark of apoptosis and used to access apoptosis [46]. Thus, our data indicated that CuSO_4_-induced nephrotoxicity involved the mitochondrial apoptotic pathway.

Copper can accumulate in the liver, kidney and brain tissues, and it is up to 17.5 μg/g kidney (wet weight) (it is equal to about 270 μM) when rats were orally administered with 200 mg/kg/day for 30 days [11]. The literature suggested that CuSO_4_ exposure at the doses of 50–1000 μM exhibited cytotoxicity in the male germ cells indicated, chicken hepatocytes, and human Caco-2 and HepG2 cells in vitro [45,47,48]. In the present study, CuSO_4_ treatment at 50–400 μM for 24 h significantly decreased the cell viability of HEK293 cells in a dose-dependent manner (Figure 5). Furthermore, our data showed that antioxidants SOD and CAT supplementation could partly inhibit CuSO_4_-induced production of ROS, the activities of caspases-9 and caspase-3, and finally inhibited CuSO_4_-induced cytotoxicity (Figure 5). These findings are indicating that oxidative stress may contribute to CuSO_4_-induced mitochondrial dysfunction. Accordingly, antioxidants that target SOD or CAT expression or activity may attenuate CuSO_4_-induced mitochondrial dysfunction and nephrotoxicity.

We further examined the role of the endoplasmic reticulum in CuSO_4_-induced nephrotoxicity. The ER stress pathway can be initiated by multiple factors, including cytotoxicity, nutrient limitation, and accumulation of unfolded or misfolded proteins [49]. GRP78 and GRP94, the ER-located chaperones, bind to transmembrane proteins such as PERK, inositol-requiring enzyme 1 (IRE1), and activating transcription factor 6 (ATF6), which all play protective roles to promote cell survival under normal physiological conditions [20,50,51,52]. PERK activation can phosphorylate eIF2α and lead to attenuation of mRNA translation [49]. ATF6 is activated via proteolysis in the Golgi apparatus, and IRE1α cleaves X-box binding protein 1 (XBP1) mRNA to form spliced XBP1, finally leading to the induction of chaperones and ER-associated degradation proteins [53]. The pro-apoptotic factor CHOP and caspase-12 are induced, then ultimately induce cell death through its downstream effectors, caspases-9 or -3 [20,50]. A recent study reported that ER stress in a mouse model was associated with CuSO_4_-induced liver damage and increased levels of GRP78, GRP94, PERK, IRE1, ATF6, ATF4, CHOP, caspase-12 mRNAs and proteins [8]. In the present study, after mice were administered orally with CuSO_4_ at 50–200 mg/kg/day for 28 days, the mRNA levels of GRP78, GRP94, CHOP, and caspase-12 significantly increased (Figure 6). Rao et al. reported that 4-PBA treatment inhibited the cyclosporine-induced increases in the mRNA expression of ER stress markers CHOP, GRP78, XBP1, and XBP1 splice in human gingival fibroblasts [54]. In another study, CuSO_4_ exposure (at 300 μM) markedly up-regulated the expression of the ER stress markers phosphorylated eIF2α and spliced XBP1 as well as DNA damage in a human hepatocyte cell line (e.g., OUMS29), which were significantly inhibited by 4-PBA [53]. Hence, these findings revealed the role of ER stress in the cytotoxicity and nephrotoxicity caused by CuSO_4_. Moreover, previous studies have shown that 4-PBA administration in mice can attenuate tunicamycin induced renal toxicity [55]. Similarly, our present study also showed that 4-PBA administration significantly improved CuSO_4_-induced renal dysfunction and pathology damage (Figure 7). In addition, the ER stress inhibitor, 4-PBA, partially inhibited CuSO_4_ exposure (at 400 μM) and tunicamycin-induced cytotoxicity and down-regulated CuSO_4_-induced increased expression of caspase-12 and CHOP mRNAs in HEK293 cells (Figure 8). It is well known that tunicamycin is an inducer of ER stress-dependent cell death [55]. Taken together, our data suggested that targeting inhibition of ER stress may be a new strategy to ameliorate CuSO_4_-induced nephrotoxicity. ER is also the target of ROS [55,56], but it is unclear whether activation of ER stress is a primary or secondary effect of CuSO_4_ exposure. Furthermore, the crosstalk between oxidative stress and ER stress in Cu exposure-induced nephrotoxicity needs further investigations.

**Figure 9:**
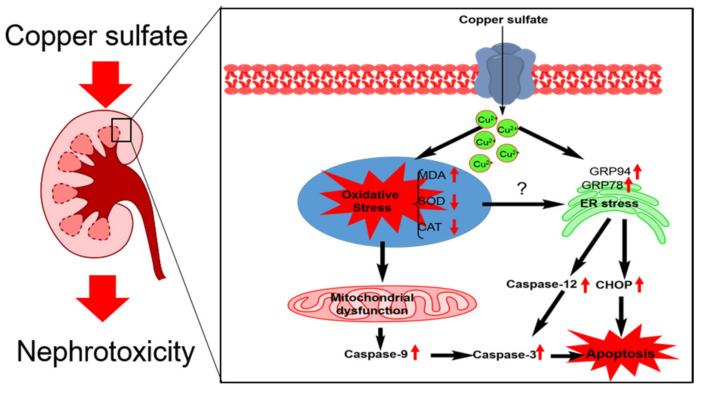
A schematic work model of CuSO_4_ exposure-induced nephrotoxicity. CuSO_4_ exposure induces oxidative stress and cascades to trigger the mitochondrial apoptotic pathway, which finally leads to nephrotoxicity in mice. Meanwhile, CuSO_4_ exposure up-regulates the expression of GRP78 and GRP94, then cascades to activate pro-apoptotic factors CHOP and caspase-12, which finally cause cell apoptosis.

In conclusion, our results reveal CuSO_4_ exposure induces oxidative stress and cascades to trigger the mitochondrial apoptotic pathway, which finally leads to nephrotoxicity in mice. We report that CuSO_4_ exposure can up-regulate ER stress, which contributes to nephrotoxicity. A proposal work model of CuSO_4_ exposure-induced nephrotoxicity is shown in Figure 9. Finally, our findings highlight that therapeutic strategies to target ER stress and oxidative stress may offer an approach for Cu exposure-induced nephrotoxicity.

## Figures and Tables

**Table 1:** Primer sequences used for quantitative real-time PCR.

Gene	Direction	Primer Sequence (5′ to 3′)
CHOP	forward	5′-AGG AGA ACG AGC GGA AAG TG-3′
	reverse	5′-GAC CAT GCG GTC GAT CAG AG-3′
GRP78	forward	5′-TCA GCA TCA AGC AAG GAT TG-3′
	reverse	5′-GCT TCA TGG TAG AGC GGA AC-3′
GRP94	forward	5′-GTC TCC CTG TGC TCT TGT GG-3′
	reverse	5′-CGT CTG GTA TGC TTG TGC CT-3′
Caspase-12	forward	5′-TGC TGG ATG GGG TTT TTG ATG-3′
	reverse	5′-TGT TCA GGA TGA AAC TCG CAC T-3′
Caspase-9	forward	5′-TGC ACT TCC TCT CAA GGC AGG ACC-3′
	reverse	5′-TCC AAG GTC TCC ATG TAC CAG GAG C-3′
Bax	forward	5′-TTC ATC CAG GAT CGA GCA GG-3′
	reverse	5′-TCC GTG TCC ACG TCA GCA AT-3′
Caspase-3	Forward	5′- TCG CAG CAT TTC TCC TAA G-3′
	Reverse	5′-GAC AAC TGG ATC GCT TGA GG-3′
GAPDH	Forward	5′-ACA GTC CAT GCC ATC ACT GCC-3′
	reverse	5′-GCC TGC TTC ACC ACC TTC TTG-3′
